# Aerosol Particle Diffusivity in the Free Molecule Regime

**DOI:** 10.1021/acs.jpca.5c00407

**Published:** 2025-06-03

**Authors:** Katerina S. Karadima, Dimitris G. Tsalikis, Vlasis G. Mavrantzas, Sotiris E. Pratsinis

**Affiliations:** † Department of Chemical Engineering, University of Patras, Patras GR-26504, Greece; ‡ Institute of Chemical Engineering Sciences (ICE−HT/FORTH), Patras GR-26504, Greece; § Particle Technology Laboratory, ETH Zurich, Zurich CH-8092, Switzerland

## Abstract

The aerosol nanoparticle (NP) diffusivity in the crossover regime from molecules to tiny (<5 nm) NPs is still in question despite the prime significance of this regime for nanotechnology as well as for aerosol fundamentals: nucleation rate, transport, coagulation, and condensation in the free molecular regime. Experiments in the past have attempted to address this regime by employing micron-sized particles and operating at low pressures to attain the large Knudsen numbers (*Kn* > 10), characteristic for this regime. However, such efforts miss the atomic level interactions between aerosol particles and surrounding gas molecules. Such interactions are dominant at the low end of the nanoscale. Here, diffusion coefficients of tiny (from 0.4 to about 7 nm in diameter) fullerene and silica particles in air are obtained by molecular dynamics (MD) simulations wherein both particles and gas molecules are considered in their full atomistic representation (force field and shape). Below 3 nm, these MD-derived diffusivities are in excellent agreement with an experimentally based equation for gas diffusivities but show systematic deviations from the classic Epstein and Stokes-Cunningham-Millikan (SCM) equations for particle diffusivity. These deviations become most pronounced as the NP size approaches that of gas molecules. Above 5 nm, the MD-derived diffusivities nicely converge to these equations. These diffusivities are compared also to other literature equations for particle diffusivity in this size regime at ambient conditions.

## Graphical abstract

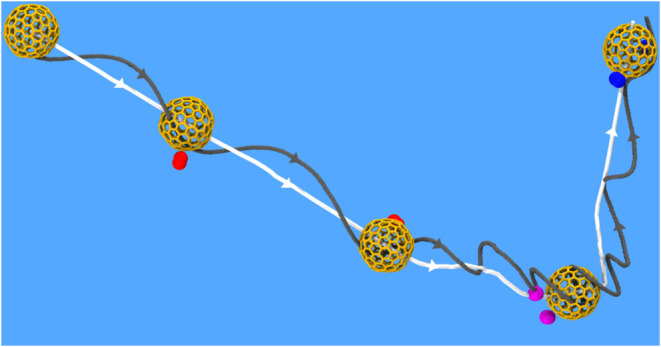

## 1 Introduction

The particle diffusivity is essential for description of aerosol transport and growth in nature as well as in industrial processes. During the last two centuries many theoretical and semiempirical equations and modifications thereof (e.g., the classical kinetic theory or the Stokes–Einstein relation) have been proposed.^1^ Among them, the Stokes-Cunningham-Millikan (SCM) equation for the diffusivity of aerosol particles is the most established one that is included in the ISO 15900:2020 standard^2^ for determining particle size distributions^3^ by differential mobility analyzers (DMAs). This equation was first parametrized by Millikan^4^ who considered micron-sized droplets at very low pressures (implying long mean free paths of the gas molecules) to reach the free molecular regime. His approach was followed by many facilitating a wide acceptance of the SCM equation. However, discrepancies were reported later when smaller particles were employed in the measurements,^5,6^ questioning the capacity of this equation to describe particle diffusivity in the free molecular regime.

For example, Scheibel and Porstendörfer^5^ reported differences close to 20% between the mobility diameter of small particles and that measured by electron microscopy (EM). Tammet^7^ first discussed the limitations of the SCM equation in the free molecular regime. He reported that interactions between gas molecules and nanosized particles cannot be captured adequately by the hard-sphere model, the foundation of the SCM equation. He distinguished emphatically between particle mass diameter and collision diameter proposing a modification of the SCM equation with an effective particle diameter to account for the gas molecule diameter and van der Waals effects.^7^ Motivated by Tammet’s distinction between mobility (or aerodynamic) diameter and particle mass diameter, Loscertales^8^ proved that the hypersonic impactor performance depends on the size treatment of nanoparticles (NPs) demonstrating that these two diameters differ for small NPs. de la Mora et al.^9,10^ modified Epstein’s^11^ equation in the free molecular regime by replacing the particle diameter with an effective one (as Tammet^7^), assuming dominant inelastic collisions over elastic ones.

Rudyak et al.^6^ reported a systematic error in NP diameters measured by DMAs compared to those by EM. In effect, the DMA estimations based on the SCM equation correlate NP diffusivities to larger particles, with the relative error increasing with decreasing particle size. Rudyak and Krasnolutski^12^ also recognized the importance of van der Waals interactions between gas molecules and particles that led them to propose modeling particles as a true collection of atoms. By considering averaged Lennard-Jones interactions, important deviations (which became more pronounced with decreasing diameter) were observed for NPs with a diameter below 20 nm.^6^

Li and Wang^13^ also argued that interactions in the nanoscale should be different from those experienced by a large particle (of micrometer-size) and introduced a reduced collision integral that accounts for both specular and diffuse scattering, weighed by a momentum accommodation function. They proposed a particle diffusivity for *d*_p_ > 1 nm by including both particle and gas molecule Lennard-Jones parameters.^14^ Jung et al.^15^ updated the most recent parametrization of the SCM equation employing also low pressures to reach high *Kn* numbers (up to 81).

Despite all these advancements and awareness of the community, a unified approach to accurately determine aerosol diffusivity *down to the tiniest of nanoparticles*, is still necessary. In specific, the crossover regime from the molecular size to that of a few nm is important for both fundamentals and applications. This regime is crucial for nanotechnology as it is there where material properties most differ from those in the bulk. In addition, in this regime nucleation largely takes place and free molecular coagulation starts. Recently, Larriba-Andaluz and Carbone^16^ reviewed several modifications of the SCM equation as well as its corrections (e.g., for nonspherical particles) pointing out its *wide margin for improvements*. They recognized the need to account for the full interatomic potential for interactions between nanoparticles highlighting the capacity of molecular dynamics (MD) simulations to improve understanding of aerosol dynamics.

Indeed MD is a powerful tool for elucidating crucial aspects of aerosol dynamics such as cluster coalescence or growth,^17^ hydrocarbon to soot growth,^18^ sintering,^19^ surface segregation^20^ and soot inception,^21^ to mention a few. Recently, Tsalikis et al.^22^ showed that by accounting for the shape and attractive-repulsive interactions of gas molecules, the gas mean free path is nearly half that given in all textbooks. By extending their study to pressures from 0.5 to 5 atm and temperatures from 100 to 3000 K, they derived a new equation for the mean free path of air as a function of temperature and pressure.^23^ Here the diffusivity of fullerenes and small silica NPs with *d*_p_ from 0.4 to 7 nm in air at ambient conditions is determined by MD simulations. Both NPs and air molecules (in stoichiometric composition) are described in their full atomistic (FA) detail. All results have been averaged over many different independent trajectories (starting from various initial configurations) to enhance sampling and reduce statistical uncertainty. These MD-derived diffusivities are compared to the most known formulas in the field.

## 2 Theory

Cunningham^24^ reported that for particles small enough compared to the surrounding gas mean free path, λ, the stick boundary condition^25^ does not apply. So he introduced the so-called slip (or Cunningham) correction factor, *C*, to the Stokes–Einstein equation for particle diffusivity:^26^

1 D=kBT3πμdpC=kBT3πμdp{1+AKn}=kBT3πμdp{1+1.632λairdp}

where *A* is the slip correction parameter and *Kn* the Knudsen number (2λ_air_ */d*_p_). Knudsen and Weber^27^ recognized that *C* is constant only for small *Kn*; for high *Kn* it should depend on *d*_p_, so they included three parameters (*A*_1_, *A*_2_, and *A*_3_) in the above equation

2 $$D=\frac{k_{B}T}{3\pi \mu d_{p}}\left\{1+\frac{2\lambda_{air}}{d_{p}}\left[A_{1}+A_{2}\exp\left(-A_{3}\frac{d_{p}}{2\lambda_{air}}\right)\right]\right\}$$

In a series of experiments, Millikan^28^ confirmed Cunningham’s^24^ argument and determined the above parameters.^4,29^ Since then, these parameters have been determined for several particle types (e.g., oil drops and polystyrene latex spheres) and mediums (e.g., air, He, etc.). Jung et al.^15^ tabulated these parameters as reported by Davies,^30^ Wahi and Liu,^31^ Allen and Raabe,^32,33^ Rader,^34^ and corrected the *C* measured by Kim et al.^35^ Their^15^ formula will be referred here as the Stokes-Cunningham-Millikan (SCM) equation

3 $$D=\frac{k_{B}T}{3\pi \mu d_{p}}\left\{1+\frac{2\lambda_{\mathrm{air}}}{d_{p}}\left[1.165+0.480\exp\left(-1.001\frac{d_{p}}{2\lambda_{\mathrm{air}}}\right)\right]\right\}$$

For spherical and rather small particles compared to λ_air_ (*Kn* > 10) and by exploring the forces exerted on them for different types of gas molecule collisions and reflections (scattering) from the particle surface, Epstein^11^ proposed the particle diffusivity

4 $$D=\frac{k_{B}T}{d_{p}^{2}}\frac{1}{\frac{2}{3}\gamma p\sqrt{\frac{2\pi m_{\mathrm{gas}}}{k_{B}T}}}$$

where *m*_gas_ is the mass of the gas molecules, *p* the pressure, and γ a factor accounting for the type of scattering (collisions) between gas molecules and particles with limiting values of 1 and 1 + π/8 for specular and diffuse scattering, respectively. The SCM equation matches Epstein’s at large *Kn* ^14^ as reported also by Millikan^4^ who used a diffuse fraction of around 0.9 to best describe his data.

Tammet^7^ modified the SCM equation by recognizing the importance of force fields. The *d*_p_ was replaced by an effective diameter, δ, namely the sum of particle and gas molecule diameters plus an extra term *h*, and two numerical factors, *f*_1_ and *f*_2_, accounting for the gas/particle masses and the collision integral Ω^(1,1)^, respectively, were included

5 D=kBT6πμδf1f2{1+λairδ[1.2+0.5⁡exp(−δλair)]}δ=rp+h+rgasf1=1+mgasmpf2=2.25(1.2+0.5)Ω(1,1)

For neutral particles, the *f*_2_ allows for a smooth transition from elastic to inelastic collisions between 1.4 and 2 nm. That way NPs and gas molecules are not treated anymore as hard spheres and the van der Waals forces are indirectly accounted for.^7^ de la Mora et al.^9^ recognized the critical role of interactions between gas molecules and small NPs (*d*_p_ < 10 nm). Based on Tammet’s concepts and Epstein’s formulation, they modified eq 4 for NPs having *d*_p_ > 2 nm by including an effective diameter that accounted also for the gas molecule diameter plus a coefficient accounting for the fraction of inelastic collisions, α^10^

6 $$D=\frac{k_{B}T}{{\left(d_{p}+d_{g,\mathrm{eff}}\right)}^{2}}\frac{3}{2p\left[1+\frac{\pi \alpha }{8}\right]}\sqrt{\frac{k_{B}T}{2\pi }\frac{1}{m_{\mathrm{gas}}}}$$

The α, known also as momentum accommodation coefficient, was inspired by Epstein’s^11^ γ (eq 4) to describe collisions of gas molecules with particles. The α was taken to be 0.91 by comparing the equation for the friction of a sphere in the free molecular regime by Friedlander^1^ and Epstein,^11^ facilitating the interpretation of available experimental data.^10^ This α value means mainly inelastic collisions, in accord with Millikan^4^ and Li and Wang.^13^

To account for van der Waals interactions between NPs and gas molecules, Li and Wang^14^ proposed a diffusivity equation for NPs having *d*_p_ > 1 nm. This includes an average reduced collision integral, Ω_avg_^(1,1)*^, namely, a weighed sum of the reduced collision integrals for diffuse, Ω_d_^(1,1)*^, and specular, Ω_s_^(1,1)*^, scattering with a momentum accommodation coefficient, φ^13,14^

7a $$\begin{array}{l} D=\frac{k_{B}T}{3\pi \mu }\frac{1}{d_{p}}{\left[1+{\left(b^{\prime }\frac{2\lambda_{\mathrm{air}}}{d_{p}}\right)}^{-1.143}\right]}^{-1/1.143} \end{array}$$

7b $$b^{\prime }=\frac{45\pi }{64}\sqrt{1+\frac{m_{\mathrm{gas}}}{m_{p}}}\frac{1}{\Omega_{\mathrm{avg}}^{\left(1,1\right)*}\Omega^{{\left(2,2\right)}^{*}}}$$

7c $$\Omega_{\mathrm{avg}}^{{\left(1,1\right)}^{*}}=\phi \Omega_{d}^{{\left(1,1\right)}^{*}}+\left(1-\phi \right)\Omega_{s}^{{\left(1,1\right)}^{*}}$$

7d $$\phi =\frac{1+0.9\mathrm{Kn}\left\{1-1/\left[1+{\left(d_{p}/5\right)}^{15}\right]\right\}}{1+\mathrm{Kn}}$$

7e $$\Omega_{d}^{\left(1,1\right)*}=1+\frac{\pi }{8}+\left[1.072+\frac{2.078}{T^{*1/4}}+\frac{1.261}{T^{*1/2}}\right]\sigma^{\prime }+\left[3.285-\frac{8.872}{T^{*1/4}}+\frac{5.225}{T^{*1/2}}\right]\sigma^{\prime 2}$$

7f $$\Omega_{s}^{\left(1,1\right)*}=1+\left[0.316+\frac{1.47}{T^{*1/4}}+\frac{0.476}{T^{*1/2}}\right]\sigma^{\prime }+\left[1.53-\frac{5.013}{T^{*1/4}}+\frac{4.025}{T^{*1/2}}\right]\sigma^{\prime 2}$$

7g $$T^{*}=\frac{k_{B}T}{\varepsilon^{\prime }}$$

7h $$\varepsilon^{\prime }=\frac{2\pi \varepsilon \sigma^{3}}{3V}$$

7i $$\sigma^{\prime }=\frac{\sigma }{d_{p}/2}$$

7j $$\varepsilon =\sqrt{\varepsilon_{g}\varepsilon_{p}}$$

7k $$\sigma =\frac{\sigma_{g}+\sigma_{p}}{2}$$

7l $$V=\frac{\mathrm{M̅}}{\rho_{s}}$$

In the above equation Ω^(2,2)*^ is the reduced collision integral of the medium viscosity, ρ_*s*_ is the density of the NPs at their melting point, *V* their molar volume and *M̅* the mean atomic mass. The introduced parameters were parametrized by temperature and van der Waals (σ and ε) parameters (reduced collision integrals) and also by *d*_p_, reproducing well Millikan’s data.^4^

Of great relevance to diffusion of tiny NPs in air is the diffusivity equation for binary gas mixtures by Fuller et al.,^36^ which is based on the rigorous Hirschfelder-Bird-Spotz equation and 308 experimental values of the diffusivities of various gases:

8 $$D=10^{-3}\frac{\sqrt{T^{3.5}\left(\frac{1}{M_{A}}+\frac{1}{M_{B}}\right)}}{p{\left[{\left(\sum_{i}V_{i,A}\right)}^{1/3}+{\left(\sum_{i}V_{i,B}\right)}^{1/3}\right]}^{2}}$$

where *M* and *V* stand for the molar mass and diffusion volume, respectively, of gases A and B, while *i* is an index running over all atoms of A or B. Equation 8 is as successful as the Chapman-Enskog equation^37^ for gas diffusivity in all textbooks today.^38^

## 3 Methods

The motion of thirteen (13) fullerene and three (3) amorphous silica NPs was examined at ambient conditions in air by MD simulations. The NP diameters ranged from about 0.4 to about 7 nm (Table S1). All fullerene particles up to C_720_ were constructed according to the C*_n_* *Fullerenes* online library,^39^ while the bigger ones were built using the general purpose open-source program *Fullerene (version 4*.*5)*.^40^ The silica particles were constructed according to Skountzos et al.:^41^ A large SiO_2_ crystal structure was built by replicating the corresponding unit cell of SiO_2_ *a*-quartz crystal^42^ thirty (30) times along each space direction. This structure was subsequently subjected to melting at high temperature (3000 K) and pressure 1 atm to produce a fully amorphous structure. This was quenched to room temperature by MD at a rate of 10 K ps^–1^. From the final structure, compact, nearly spherical NPs of the desired diameter and with the correct stoichiometry were cut out. These NPs were further equilibrated at 298 K under constant volume to relax mostly their surface structure.

With these NPs, MD simulations were performed using cubic cells filled with air molecules (79 mol % N_2_ and 21 mol % O_2_) corresponding to a NP volume fraction of 10^–5^ (except for the very tiny NPs with subnano diameter where the volume fraction was even smaller, 10^–6^). For the fullerenes, nonbonded interactions were described by the Lennard-Jones potential as proposed by Girifalco et al.,^43^ while for the bonded ones, the general-purpose DREIDING force field^44^ was chosen (Tables S2–S6). For the silica NPs, the Beest-Kramer-van Santen (BKS) potential^45^ using the Lennard-Jones potential reparametrization of Barbier et al.^46^ was employed, while nitrogen and oxygen atoms were treated (Table S2) according to the two-site model of Tsolou et al.^47^ and Karadima et al.^48,49^

All NP simulations were performed in the isothermal–isobaric *(NpT*) statistical ensemble at 298.15 K and 1 atm while several of them (about 70%) were carried out in the *NVE* ensemble also. They lasted for several hundreds of ns (even up to μs in some cases, depending on NP size and type) to ensure that normal (Fickian) diffusion is well reached to reliably extract the diffusion coefficient from the mean square displacement of the NP center of mass. In the simulations, van der Waals interactions were considered up to 16 Å, and for special interactions as well as Lennard-Jones pair coefficients between different types of atoms, the rules as defined by the DREIDING force field^44^ were followed for the fullerene NPs. For the silica NPs both the van der Waals and the electrostatic interactions were considered up to 12 Å, while the electrostatic interactions beyond this distance were calculated with the particle–particle particle-mesh (PPPM) method.^50^ The simulations were conducted with the LAMMPS open-source software.^51,52^ Atomic positions were stored every 100 ps, and the mean square displacement (MSD) was calculated over the equilibrated parts of MD trajectories by the technique of multiple time origins^53^ to obtain smoother MSD. For each NP, the simulations were repeated (typically 12–24 times) starting from an entirely different configuration of atomic positions and/or velocities in order to decrease the statistical uncertainty of the extracted results. The resulting MSD were then averaged over all MD trajectories accumulated for that NP, and the corresponding *D* was computed from the slope of the MSD vs time *t* line, after the non-Fickian regime. The coefficient of determination, *R* ^2^, and the diffusivity were obtained by fitting the MSD vs t with MSD = 0 + 6·*D*·*t* ^1^ (Table S1) for all fullerene and silica NPs as shown in Figures S6–S9 (broken lines). The nearly overlapping MSD evolutions for both fullerene and silica NPs (Figures S6 and S9, respectively) at 2 and 3 nm indicate the limited effect of particle composition on *D* at these particle sizes.

## 4 Results and Discussion

The employed force field for air^47^ was validated by calculating the density and diffusion coefficient at 300 K and 1 atm and comparing with experimental measurements and/or well-accepted theoretical predictions for dry air.^22^ The density was estimated to be 1.173 ± 0.01 kg/m^3^, in agreement with the experimentally measured value of 1.177 kg/m^3^ at the conditions of the simulations.^56^ The MD-derived diffusion coefficient of air was 0.214 ± 0.005 cm^2^/s, which also agrees with the literature values of 0.221 and 0.204 cm^2^/s derived theoretically according to Poling et al.^57^ and Cussler,^38^ respectively. It also agrees with the value of 0.208 ± 0.001 cm^2^/s based on viscosity measurements.^58^

Accounting for the force field and shape of both gas molecules and particles reveals a variety of such collision types that had not been considered before. For example, Tsalikis et al.^22^ showed that gas molecules undergo grazing, heads-on (ballistic), multibody and spurious collisions that result in orbiting trajectories as had been envisioned by Hirschfelder et al.^59^ All these types of collisions have been observed also here between gas molecules and NPs. In Video V1, one can see a C_20_ fullerene (diameter = 0.4 nm) to move rather slowly while one N_2_ molecule moves with its characteristic translational and rotational motion^22^ to exhibit a grazing collision with the NP (Figure 1a) while in Video V2 nearly a head-on (ballistic) collision of such a N_2_ molecule takes place with the fullerene particle (Figure 1b).

**Figure 1:**
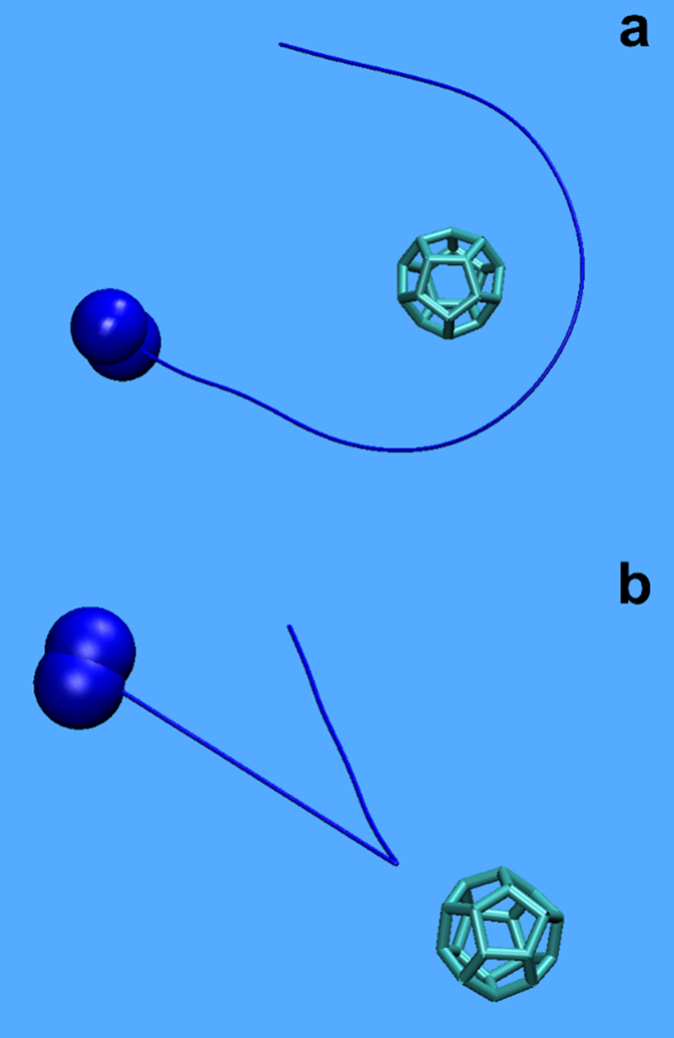
Grazing (a) and nearly head-on (b) collisions between a N_2_ molecule and a C_20_ fullerene NP in relative coordinates as depicted by the corresponding V1 and V2 videos in the Supporting Material. In both cases, the line (bold blue) shows the trajectory of the N_2_ center of mass.

Accounting for the detailed potential and shape of gas molecules, significantly enhances collisions way above those predicted by the kinetic theory of gases, mainly due to the attractive part of the Lennard-Jones potential.^22^ This is also the case for the interactions of the gas molecules with the NPs reducing their MSD. Figure 2 illustrates the tumbling motion of a fullerene NP having 1.2 nm diameter as it diffuses through air. The trajectory of the NP’s center of mass is depicted by a white line, highlighting the translational movement of the NP, while the gray line shows its rotational motion by tagging one of its carbon atoms. The NP alters its direction due to multibody and orbiting collisions^22^ by air molecules and even their transient “adsorption” onto the NP. The detailed NP motion is depicted in Video V3.

**Figure 2:**
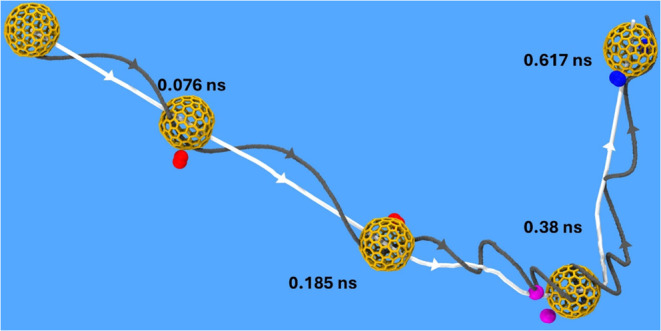
Trajectory of a 1.2 nm fullerene NP (orange) diffusing in air as depicted by Video V3 in the Supporting Material. The paths of the NP center of mass (white line) and of an atom on the NP surface (gray line) depict its translational and rotational motion, respectively.

Figure 3 shows the MD-derived diffusion coefficients *D* (symbols) of fullerene (circles) and silica NPs (triangles) as a function of their *d*_p_. Within the simulation variability (error bars), equally sized fullerene and silica NPs have the same *D* for these sizes (2 and 3 nm in *d*_p_). The *D* of small fullerenes (*d*_p_ < 3 nm) closely follows eq 8 of Fuller et al.^36^ that is based on experimental measurements of binary gas mixtures further validating the present simulations. Table S9 shows exemplary calculations of *D* for C_20_ and C_60_, according to eq 8. The deviation for larger fullerenes is expected, as eq 8 has been developed from measurements with smaller gas molecules. For these large fullerene and silica particles, the MD-derived *D* asymptotically approach the well-established SCM and Epstein eqs (eqs 3 and 4, respectively). The hydrodynamic contribution to NPs diffusivity due to system size effects^54,55^ is typically 3 orders of magnitude smaller than the MD-extracted ones here (Table S7) for the system sizes employed here. The hydrodynamic contribution is therefore neglected. Additional simulations conducted for two fullerene NPs and one silica NP with diameters 0.7, 1.2, and 2 nm, respectively, with a simulation cell volume thrice larger than that used for the diffusivities in Figure 3 hardly differ from them (Figures S1 and S2). To investigate the impact of potential contributions from the barostat or the thermostat of the *NpT* ensemble on the MD-extracted diffusivities, additional simulations were carried out in the microcanonical (*NVE*) ensemble. These simulations were performed on selected fully relaxed configurations of both fullerene (*d*_p_ = 0.4, 0.5, 0.6, 0.7, 1.2, 2.0, and 7.0 nm) and silica nanoparticles (*d*_p_ = 1.5, and 2 nm). In all cases, the diffusivities obtained from the *NVE* ensemble of the MD trajectories (Figures S3–S5) were in agreement with those from the *NpT* ones (Figure 3).

Most importantly and in agreement with Tammet,^7^ de la Mora et al.,^10^ Rudyak et al.^6^ and Li and Wang,^13^ for *d*_p_ < 5 nm the MD-predicted *D* deviate from the *D* obtained from the SCM and Epstein equations. These deviations are becoming more pronounced as the NP diameter approaches the size of surrounding gas molecules (about 0.4 nm). Quite likely this is due to the fact that both SCM and Epstein equations neglect the details of gas–NP collisions (grazing, spurious, orbiting and even multibody ones), that become most important as the particle sizes become comparable to that of gas molecules.

**Figure 3:**
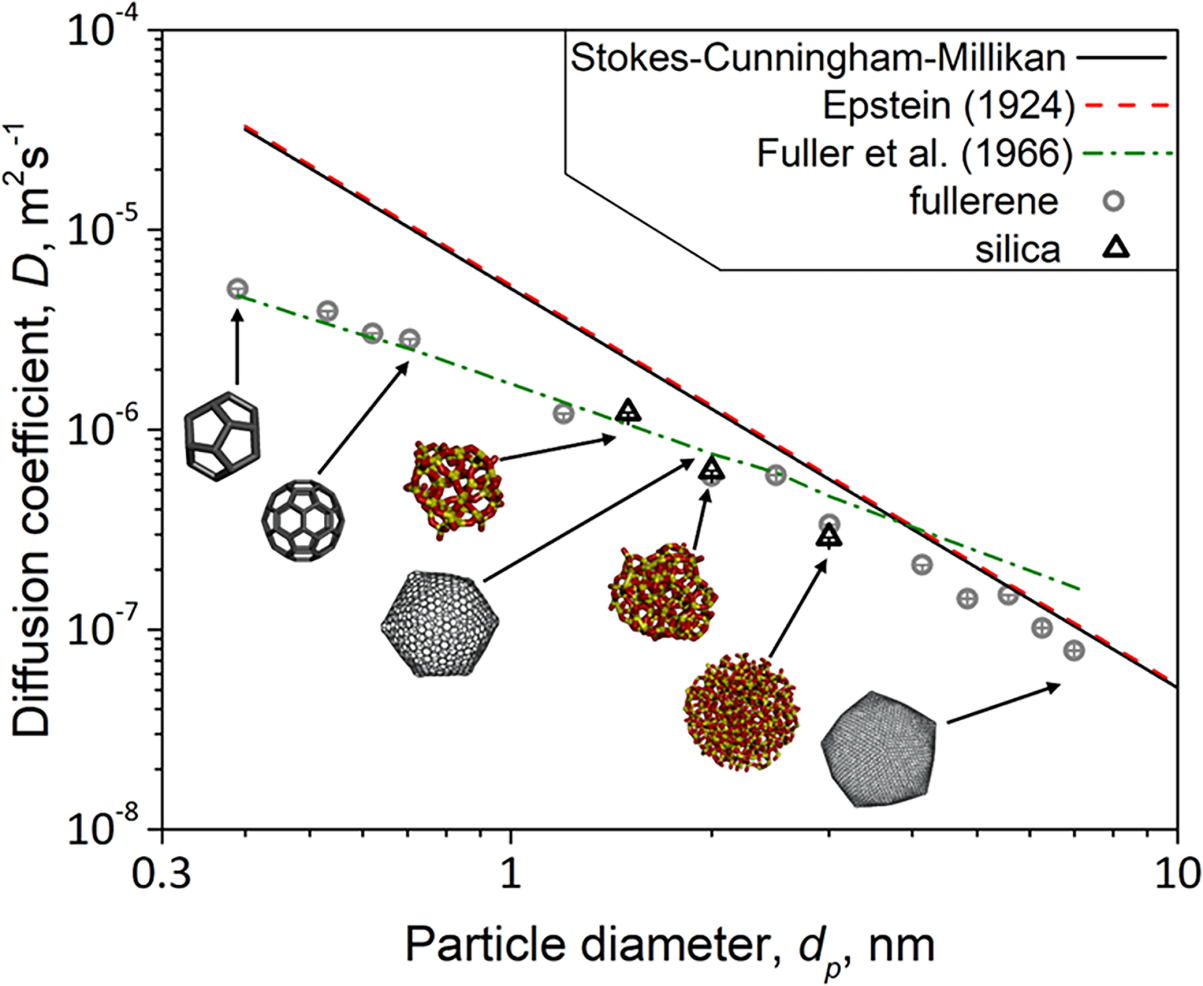
MD-obtained diffusion coefficients of fullerene (circles) and silica (triangles) NPs as a function of their *d*_p_ along with those from the Stokes-Cunningham-Millikan (solid black line) and Epstein^11^ (broken red line) equations (that totally overlap in this size regime) as well to that (dot-broken green line) by Fuller et al.,^36^ eq 8. Inset particle schematics are not to scale. The errors in the MD-obtained diffusion coefficients are smaller than the symbol size. The schematics were produced with the free-distributed software BIOVIA Discovery Studio Visualizer.^60^

To shed more light on the discrepancy observed between the *D* values from the SCM equation and the MD simulations, it is noted that the SCM equation considers both particles and gas molecules as rigid bodies. In contrast, in the MD simulations the employed force field accounts for the actual shape and all possible interactions between gas molecules and NPs that differ considerably from the notion of rigid bodies. This is critical for interactions between gas molecules and the tiniest NPs: the smaller the NP, the larger is the fraction of its atoms that interact with the surrounding gas molecules. For example, for a 0.4 nm fullerene NP all of its atoms interact with gas molecules (via the Lennard-Jones potential). As NP size increases, the impact of these interactions diminishes as the force field of gas molecules affects less and less the motion of NP and its transport (diffusive) properties, resulting in the observed convergence of the MD-obtained *D* to the SCM equation at *d*_p_ > 5 nm (Figure 3).

### 4.1 Comparison with Literature Equations for *D*

Next, it is explored how well the MD-obtained diffusivities can be described by prior equations. Figure 4 shows that eq 5 (dot broken blue line) by Tammet^7^ overpredicts the MD-derived *D* for the smallest fullerene and silica NPs. However, as the particle size increases (e.g., for *d*_p_ > 2.5 nm), the contributions from *f*_1_ and *f*_2_ in eq 5 become less significant (Figure S10), bringing it closer to the MD-obtained *D*.

**Figure 4:**
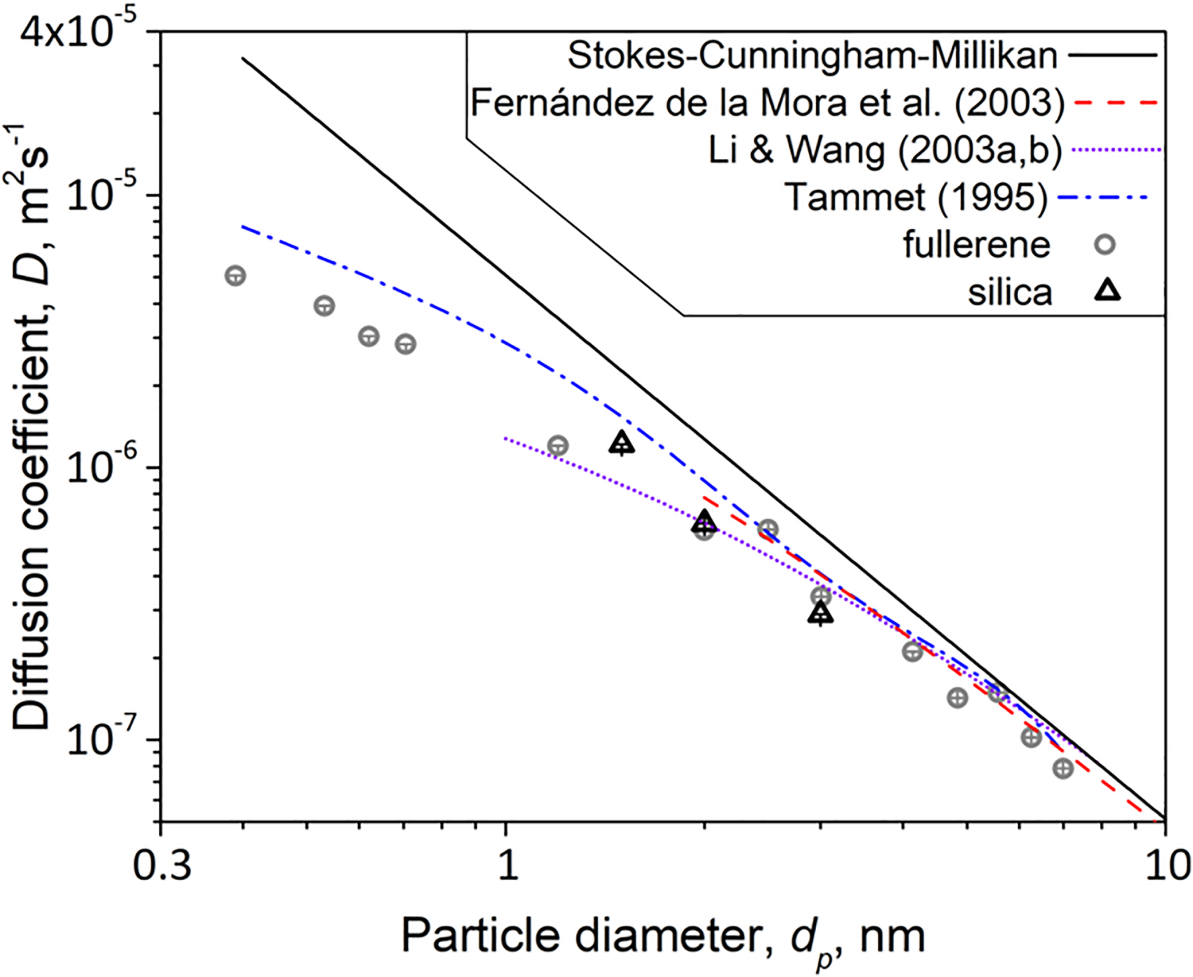
MD-derived diffusion coefficients for fullerene (circles) and silica (triangles) NPs as a function of their diameter at 298.15 K. The solid black line corresponds to the predictions of the diffusion coefficient according to the Stokes-Cunningham-Millikan’s eq 3. The broken red line stands for eq 6 by de la Mora et al.^10^ The predictions by eq 5 (dot-broken blue line) from Tammet^7^ and by eq 7 (dotted purple line) from Li and Wang^13,14^ are shown also.

Figure 4 shows also that the MD-derived *D* are followed closely by eq 6 in its regime of validity, i.e., NPs with *d*_p_ > 2 nm.^10^ The good performance of eq 6 can be understood when its underlying assumptions are examined. It is in essence an Epstein-based equation that treats both NP and gas molecules as rigid bodies with, however, the important modification that the particle diameter is replaced by an effective diameter accounting for the presence of colliding gas molecules. The value of the momentum accommodation coefficient suggested by de la Mora et al.^10^ was 0.911, signifying the dominant role of inelastic collisions between gas molecules and NPs. Clearly, as the NP diameter increases, eq 6 approaches also the Epstein and SCM ones.

The MD-predicted diffusion coefficients are compared also with those from eq 7 ^13,14^ that was proposed for *d*_p_ > 1 nm. For the first approximation of the reduced collision integrals in eq 7, the σ and ε parameters were estimated following Rudyak et al.^6^ and Li and Wang^14^ for SiO_2_ particles. More specifically, σ and *ε* were calculated from the melting point, *T*_m_, of SiO_2_ and its density there,^59^ *T*_m_ = 1713 °C^61^ and ρ_s_ = 2.52 g cm^–3^.^62^ The MD-obtained *D* are in excellent agreement with eq 7 in its regime of validity (*d*_p_ > 1 nm).

Worth noting is that Tammet^7^ described an additional transition regime for the type of collisions that dominate aerosol dynamics below a critical diameter, where elastic collisions again dominate to match those of gas molecules.^63^ He probably thought that at these very small NPs (with *d*_p_ less than 1–2 nm), aerosol particles start behaving like rigid bodies as dictated by kinetic theory for gases. Nevertheless, Tammet^7^ correctly noted that the cross section for inelastic collisions exceeds that of elastic ones so his effective diameter δ captures the effect of the force field and polyatomic nature of gas molecules (that is crucial for the tiny NPs), but decays with increasing particle size.

## 5 Conclusions

Diffusion coefficients for tiny fullerene and silica NPs in air under infinitely dilute conditions, at room temperature and pressure from detailed MD simulations are reported here accounting for the force field and true atomic structure of gas molecules and NPs. Due to strong interactions between NPs and gas molecules, significant deviations are recorded for tiny (<5 nm) aerosol NPs between their MD-estimated diffusion coefficients and those from the well-accepted Epstein^11^ and Stokes-Cunningham-Millikan equations, up to about an order of magnitude as the NP size approaches that of gas molecules, about 0.4 nm. For particles below 2 nm, the MD-derived diffusivities are in good agreement with the Fuller et al.^36^ correlation based on experimental measurements of binary gas mixtures. For particles larger than 2 nm, the corresponding diffusivity equations by Tammet,^7^ de la Mora et al.^10^ and Li and Wang^14^ follow quite closely the MD predictions, with that by de la Mora et al.^10^ slightly closest. Despite the fact that the latter is based on a rigid body model, as described by Epstein,^11^ by replacing the particle diameter by an effective one that accounts for inelastic collisions through an accommodation coefficient allows for a rather adequate description of the diffusivity. The equation by Tammet,^7^ on the other hand, overestimates the diffusivity of the tiniest NPs (diameter less than 2 nm). The Li and Wang^14^ equation considering the impact of force field, is in excellent agreement with the MD-predicted *D* in its regime of validity (*d*_p_ > 1 nm) even though it does not account explicitly for the gas molecule diameter, further validating the present analysis.

## Supplementary Material









## Notes

Characteristics of the NPs and the coefficients of determination of the fitted lines applied on the time evolution of the fullerene and silica NPs MSD curves (Table S1) and the force field parameters employed here (Tables S2–S6), calculations of the diffusion coefficient correction for system-size effects (Table S7), the mean square displacement of fullerene and silica NPs as a function of time together with the mean square displacement sections used to extract the diffusion coefficients in base case simulation cell volume, and triple simulation cell volume of the base case (Figure S1), comparison between the diffusivities predicted from the base case simulation cell volume and the triple simulation cell volume of the base case (Figure S2), the mean square displacement of silica (Figure S3) and fullerene (Figure S4) NPs as a function of time together with the mean square displacement sections used to extract the diffusion coefficients in *NpT* and *NVE* ensemble, diffusion coefficients for fullerene and silica NPs as calculated by *NpT* and *NVE* simulations (Table S8), comparison between the *NpT-* and *NVE*-predicted diffusivities (Figure S5), calculations of diffusivity coefficients for C_20_ and C_60_ fullerenes, according to eq 8 by Fuller et al.^36^ (Table S9), the mean square displacement of fullerene and silica NPs as a function of time together with the mean square displacement sections used to extract the diffusion coefficients in *NpT* ensemble (Figures S6–S9), the diffusivity from eq 6 by Tammet^7^ with and without the factors *f*_1_ and *f*_2_ (Figure S10) (PDF)
Grazing collision between a gas molecule and a fullerene particle with diameter of 0.4 nm (Video V1) (MP4)
Head-on collision between a gas molecule and a fullerene particle with diameter of 0.4 nm (Video V2) (MP4)
Trajectory of a 1.2 nm fullerene NP diffusing in air together with selected air molecules colliding with the NP (Video V3) (MP4)

## Abbreviations

BKS: Beest-Kramer-van Santen
DMA: differential mobility analyzer
ISO: international organization for standardization
MD: molecular dynamics
MSD: mean square displacement
NP: nanoparticle
PPPM: particle–particle particle-mesh
SCM: Stokes-Cunningham-Millikan

## Nomenclature

A: slip correction parameter
*A*_1_, *A*_2_, *A*_3_: parameters included in the slip correction factor definition
b’: parameter to account for the transition regime according to Li and Wang
C: slip (or Cunningham) correction factor
D: coefficient of diffusivity (m^2^ s^–1^)
*d*_p_: particle diameter (m)
*d*_*g*,*eff*_: effective diameter of gas molecule (m)
*f*_1_: factor accounting for the gas/particle masses according to Tammet
*f*_2_: factor accounting for the collision integral and the elastic-specular, and inelastic-diffuse collisions according to Tammet
h: Tammet’s extra distance term (m)
*k*_*B*_: Boltzmann constant (J K^–1^)
Kn: Knudsen number
M̅: mean atomic mass of the particle (kg mol^–1^)
*M*_*A*_, *M*_*B*_: molar mass of the two components *A* and *B* of a mixture in gas phase (kg mol^–1^)
*m*_*gas*_: gas molecule mass (kg)
*m*_*p*_: particle mass (kg)
p: pressure (Pa)
*r*_*gas*_: gas molecule collision radius (m)
*r*_*p*_: particle radius (m)
T: temperature (K)
T*: modified reduced temperature according to Li and Wang
V: effective volume of the particle per molecule (m^3^ mol^–1^)
*V*_*i*,*A*_, *V*_*i*,*B*_: diffusion volumes according to Fuller et al. of the two components *A* and *B* of a mixture in gas phase, with *i* an index running over all atoms of *A* or *B* (m^3^)

## Greek Letters

*α*: momentum accommodation coefficient according to Fernández de la Mora
γ: Epstein’s factor accounting for the type of scattering among collisions
δ: Tammet’s effective diameter (m)
*ε*: well depth of Lennard-Jones potential (J mol^–1^)
*ε’*: modified well depth of Lennard-Jones potential according to Li and Wang (J mol^–1^)
*ε*_*g*_: well depth of Lennard-Jones potential for the gas molecule (J mol^–1^)
*ε*_*p*_: well depth of Lennard-Jones potential for the constituent atom or molecule of the particle (J mol^–1^)
λ_*air*_: mean free path of air (m)
*μ*: viscosity of the medium (Pa s)
π: pi constant
ρ_*s*_: mass density of the particle (kg m^3^)
*σ*: van der Waals diameter (m)
*σ’*: reduced collision diameter
σ_*g*_: self-collision diameter of gas molecule (m)
σ_*p*_: self-collision diameter of the constituent atom or molecule of the particle (m)
φ: momentum accommodation coefficient according to Li and Wang
Ω^(1,1)^: collision integral
Ω_*avg*_^(1,1)^*^^: average reduced collision integral
Ω_*d*_^(1,1)^*^^: reduced collision integral for diffuse scattering
Ω_*s*_^(1,1)^*^^: reduced collision integral for specular scattering
Ω^(2,2)^*^^: reduced collision integral of the medium viscosity
