# Supplementary material for: Aerosol Particle Diffusivity in the Free Molecule Regime
Source: J Phys Chem A. 2025 Jun 3;129(23):5127–36. doi: 10.1021/acs.jpca.5c00407 (PMC12169690; doi:10.1021/acs.jpca.5c00407)
Supplement: Supplementary file 1 [file jp5c00407_si_001.pdf]

SUPPORTING INFORMATION

Aerosol Particle Diffusivity in the Free Molecule Regime

Katerina S. Karadima,[1–3] Dimitris G. Tsalikis,[3] Vlasis G. Mavrantzas,[1–3] Sotiris E. Pratsinis[3*]*

[1]Department of Chemical Engineering, University of Patras, Patras, GR 26504, Greece

[2]Institute of Chemical Engineering Sciences (ICE–HT/FORTH), Patras, GR 26504, Greece

[3]Particle Technology Laboratory, ETH Zurich, Zurich, CH 8092, Switzerland

*Email: vlasis@chemeng.upatras.gr and pratsinis@ethz.ch

S1. SIMULATION DETAILS AND FORCE FIELD

Table S1. Employed nanoparticles and their characteristics along with the coefficient of determination, R^2, of the fitted lines, $MSD = 0 + 6 \cdot D \cdot t^1$, applied on the MD-obtained time evolution of the fullerene and silica NPs mean square displacement (MSD) in Figures S1-S4.

No	Type	Composition, # of atoms	d_p, nm	R^2, -
1	fullerene	20 *C*	0.39	0.997
2	fullerene	36 *C*	0.53	0.946
3	fullerene	48 *C*	0.62	0.998
4	fullerene	60 *C*	0.71	0.988
5	fullerene	180 *C*	1.20	0.987
6	fullerene	500 *C*	2.04	0.826
7	fullerene	720 *C*	2.45	0.969
8	fullerene	1140 *C*	3.00	0.983
9	fullerene	2160 *C*	4.14	0.976
10	fullerene	2960 *C*	4.84	0.983
11	fullerene	3920 *C*	5.57	0.989
12	fullerene	4940 *C*	6.26	0.943
13	fullerene	6180 *C*	7.00	0.975
14	silica	42 *Si* & 84 *O*	1.5	0.996
15	silica	100 *Si* & 200 *O*	2.0	0.981
16	silica	331 *Si* & 662 *O*	3.0	0.991

Table S2. Parameters of the 12-6 Lennard-Jones, $U_{LJ}(r) = 4\varepsilon\left[\left(\frac{\sigma}{r}\right)^{12} - \left(\frac{\sigma}{r}\right)^{6}\right]$, and the Coulomb potential, $U_C(r) = \frac{1}{4\pi\varepsilon_0}\frac{q_1 q_2}{r}$, describing contributions to the potential energy function due to non-bonded (van der Waals and electrostatic) interactions.

**Atom type**	***σ*** **(Å)**	***ε*** **(kcal mol^{-1})**	***q(e)***
C	3.4681	0.06626	0
N	3.31	0.071	0
O (air)	3.09	0.089	0
Si	0	0	2.4
O (silica)	2.99	0.4723	-1.2
Si- O (silica)	1.53	26.3642	-

Table S3. Parameters of the harmonic potential, $U_b(l) = k_b(l - l_0)^2$, describing contributions to the potential energy function due to bond stretching.

**Bond type**	**l_0 (Å)**	**k_b (kcal mol^{-1} Å^{-2})**
C-C	1.39	525
N-N	1.10	450
O-O	1.21	450

Table S4. Parameters of the harmonic potential, $U_a(\theta) = k_a(\theta - \theta_0)^2$, describing contributions to the potential energy function due to bond angle bending.

**Angle type**	**θ_0 (°)**	**k_a (kcal mol^{-1} rad^{-2})**
C-C-C	120	50

Table S5. Parameters of the torsional potential $U_t(\varphi) = k_t[1 + \cos(n\varphi - d)]$, describing contributions to the potential energy function due to dihedral angles.

**Dihedral type**	***d*** **(°)**	***n***	**k_t (kcal mol^{-1})**
C-C-C-C	-180	2	3.125

Table S6. Parameters of the harmonic potential $U_i(\varphi) = k_i(\varphi - \varphi_0)^2$ describing contributions to the potential energy function due to improper dihedral angles.

**Angle type**	**φ_0 (°)**	**k_i (kcal mol^{-1} rad^{-2})**
C-C-C	0	20

S2. SIMULATION VALIDATION

The diffusivity, accounting for periodic boundaries, D_{PBC} is equal to:[54,55]

$$D_{\mathrm{PBC}} = D_{MD} + \frac{k_B T \xi}{6\pi\eta L}$$

with D_{MD}, denoting the translational diffusivity, T, the temperature, η the medium viscosity, L the simulation cell length, and ξ a numerical constant equal to ξ = 2.837297. For the system sizes employed here, the correction term, $D_{correction} = k_B T \xi / 6\pi\eta L$, by Dünweg and Kremer,[54] as well as by Yeh and Hummer[55] is 3-4 orders of magnitude smaller than the diffusion coefficients, D_{MD}, calculated here (Table S7). As such, the contribution from hydrodynamic interactions due to the finite size of the cells is practically insignificant. This is primarily due to the relatively long length of the employed simulation cells, L = 31 – 262 nm in comparison to that of the diffusing particles.

Furthermore, to ensure the complete absence of system-size effects, additional simulations for representative fullerene and silica NPs were conducted, where the volume of the simulation cell was tripled at the same temperature and pressure, increasing accordingly the population of air molecules in the cell. For all NPs the resulting diffusion coefficients agree well with those from the MD simulations with the original cell sizes (Figures S1 and S2).

Table S7. The diffusion coefficient correction for system-size effects[54,55] $D_{correction} = k_B T \xi / 6\pi\eta L$, ξ = 2.837297, η = 1.827 × 10^{-5} Pa s, for fullerene and silica NPs with simulation cell length, *L*, compared to the diffusion coefficient obtained from present MD simulations, D_{MD}.

d_p, nm	Type	*L*, nm	$D_{correction}$, m^2s^{-1}	D_{MD}, m^2s^{-1}	D_{PBC}, m^2s^{-1}
0.39	fullerene	31	1.09×10^{-9}	5.07×10^{-6}	5.07×10^{-6}
1.20	fullerene	44.2	7.63×10^{-10}	1.20×10^{-6}	1.20×10^{-6}
4.14	fullerene	155	2.18×10^{-10}	2.11×10^{-7}	2.11×10^{-7}
6.26	fullerene	234	1.44×10^{-10}	1.02×10^{-7}	1.02×10^{-7}
7.00	fullerene	262	1.29×10^{-10}	7.85×10^{-8}	7.86×10^{-8}
1.5	silica	64.8	5.20×10^{-10}	1.22×10^{-6}	1.22×10^{-6}
2.0	silica	64.8	5.20×10^{-10}	6.24×10^{-7}	6.24×10^{-7}
3.0	silica	67.1	5.02×10^{-10}	2.89×10^{-7}	2.89×10^{-7}

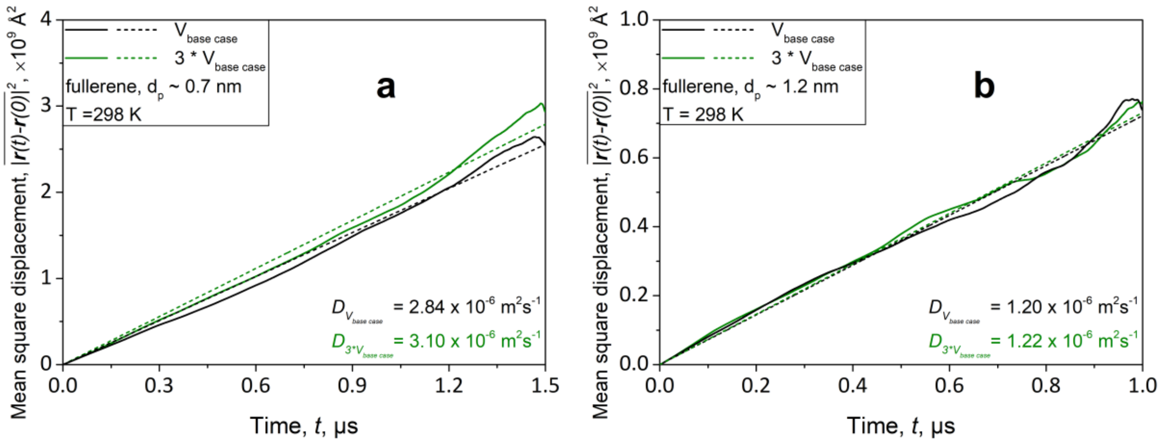

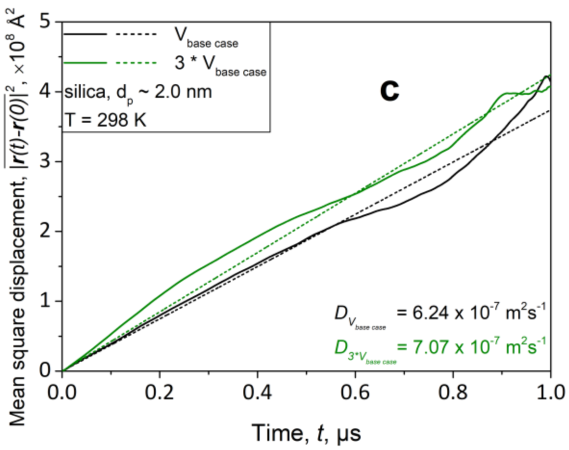

Figure S1. Mean square displacement (MSD, solid lines) as a function of time for two fullerene NPs with diameter a) 0.7, and b) 1.2 nm as well as for a silica NP c) with diameter 2.0 nm from fully atomistic (FA) MD simulations with base case simulation cell volume (black line), and thrice that volume (green line). The dotted lines correspond to fitting the above MD-obtained data with $MSD = 0 + 6 \cdot D \cdot t$.

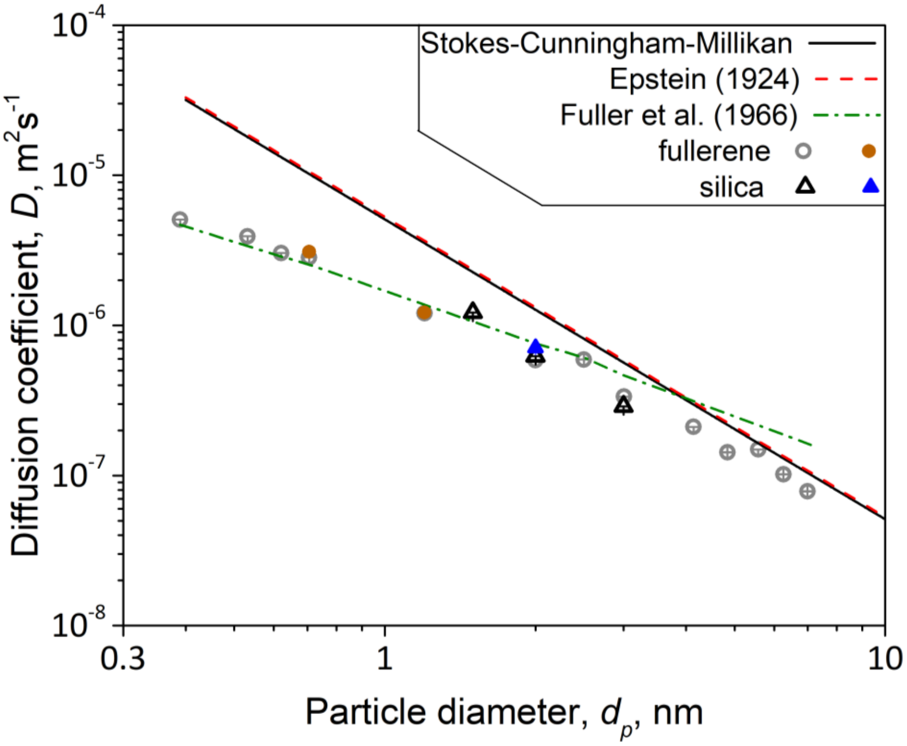

Figure S2. MD-obtained diffusion coefficients of fullerene (circles) and silica (triangles) NPs as a function of their d_p along with those from the Stokes-Cunningham-Millikan (solid black line) and Epstein (broken red line) equations (that totally overlap in this size regime) as well to that by Fuller et al. (dot-broken green line) and comparison between results obtained by employing the base cell volume (open symbols) and thrice that volume (filled symbols). The errors in the diffusion coefficients are smaller than the symbol size.

The presence of the barostat is known to influence the microscopic behavior of dilute gases.[22] Though, the macroscopic properties, such as transport ones, should be similar in equivalent ensembles. To further examine this, we have conducted additional simulations for most of the NPs. In specific, we carried out NVE simulations for the fullerene NPs with diameter 0.4, 0.5, 0.6, 0.7, 1.2, 2 and 7 nm and the silica NP with diameter 1.5 and 2 nm. To this, fully equilibrated configurations from the *NpT* simulations at the same conditions (T = 298 K and p = 1 atm) were selected and used as initial configurations in the microcanonical (*NVE*) ensemble. During these *NVE* simulations, the system's temperature, T, and pressure, p, were $T = 298 \pm 2$ K and $p = 1 \pm 0.2$ atm, respectively, in agreement with those in our earlier *NpT* simulations. The protocol utilized to calculate the NPs' diffusivity in the *NVE* simulations was identical to that used in the *NpT* ones, i.e., configurations were stored every 100 ps and were used to calculate the mean squared displacement (MSD) of the NPs. The MSDs from the *NVE* simulations were systematically averaged, and from the slope of the average MSD with time, the nanoparticle diffusivity was extracted. The MSD results obtained, as summarized in Figures R3 (silica) and R4 (fullerenes), agree well with those obtained via the MD simulations conducted in the *NpT* ensemble.

The extracted diffusivities from the MSD are reported in Table R2 and Figure R5. Overall, the maximum deviation is ~14% observed only for the fullerene NP with d_p = 0.5 nm. For all other NPs the discrepancies between the *NpT*- and *NVE*-predicted diffusivities are less than 10.4% and lie within the variation of the original simulations. The *NVE*-predicted diffusivities in Fig. R5 largely overlap with the corresponding NPT-predicted diffusivities.

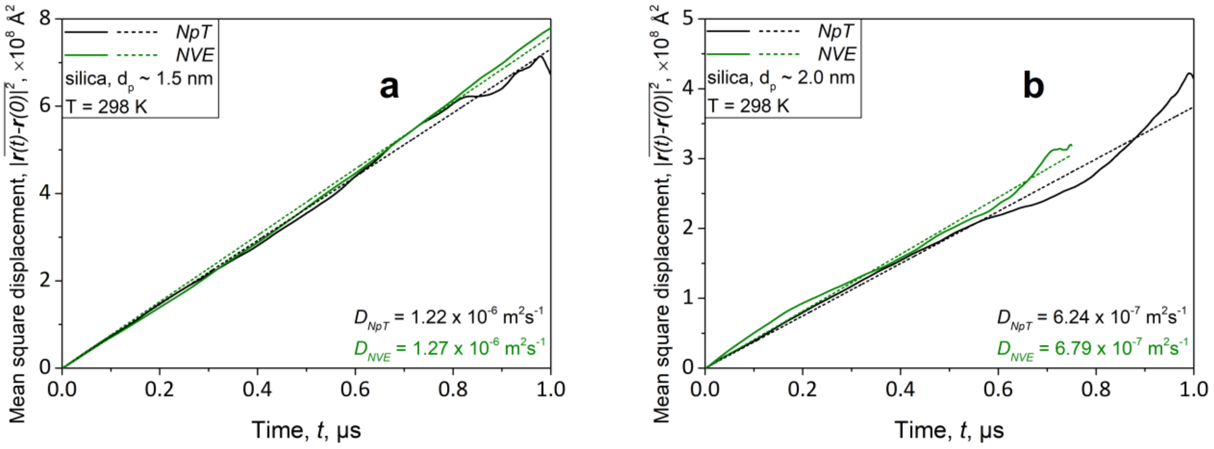

Figure S3. Mean square displacement (MSD) as a function of time for silica NPs with diameters, a) 1.5, and b) 2 nm, from fully atomistic (FA) molecular dynamics (MD) simulations in *NpT* (black line), and *NVE* ensemble (green line). The broken lines correspond to fitting the above MD-obtained data with $MSD = 0 + 6 \cdot D \cdot t$. The diffusivities from both *NpT* and *NVE* ensembles are nearly identical (insets, Table S8 also).

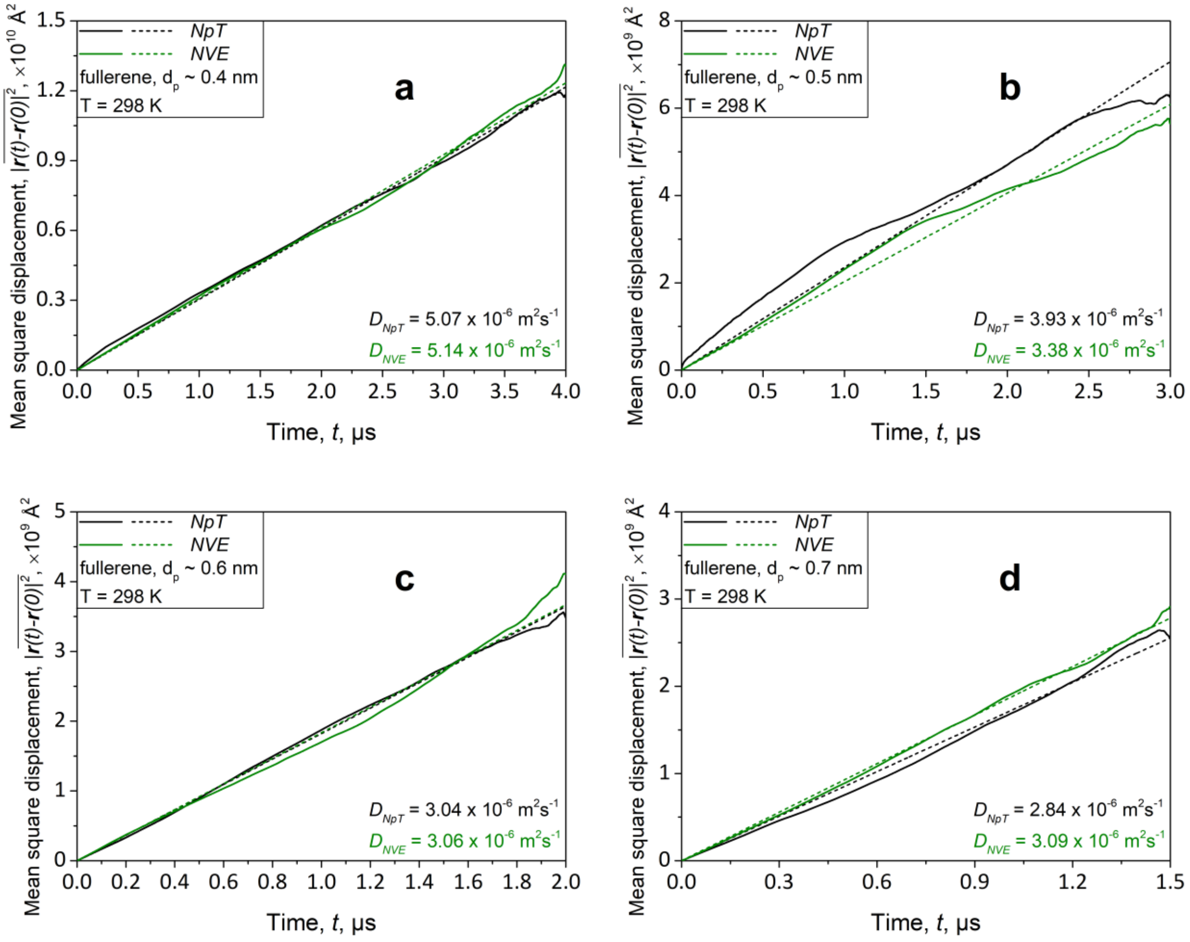

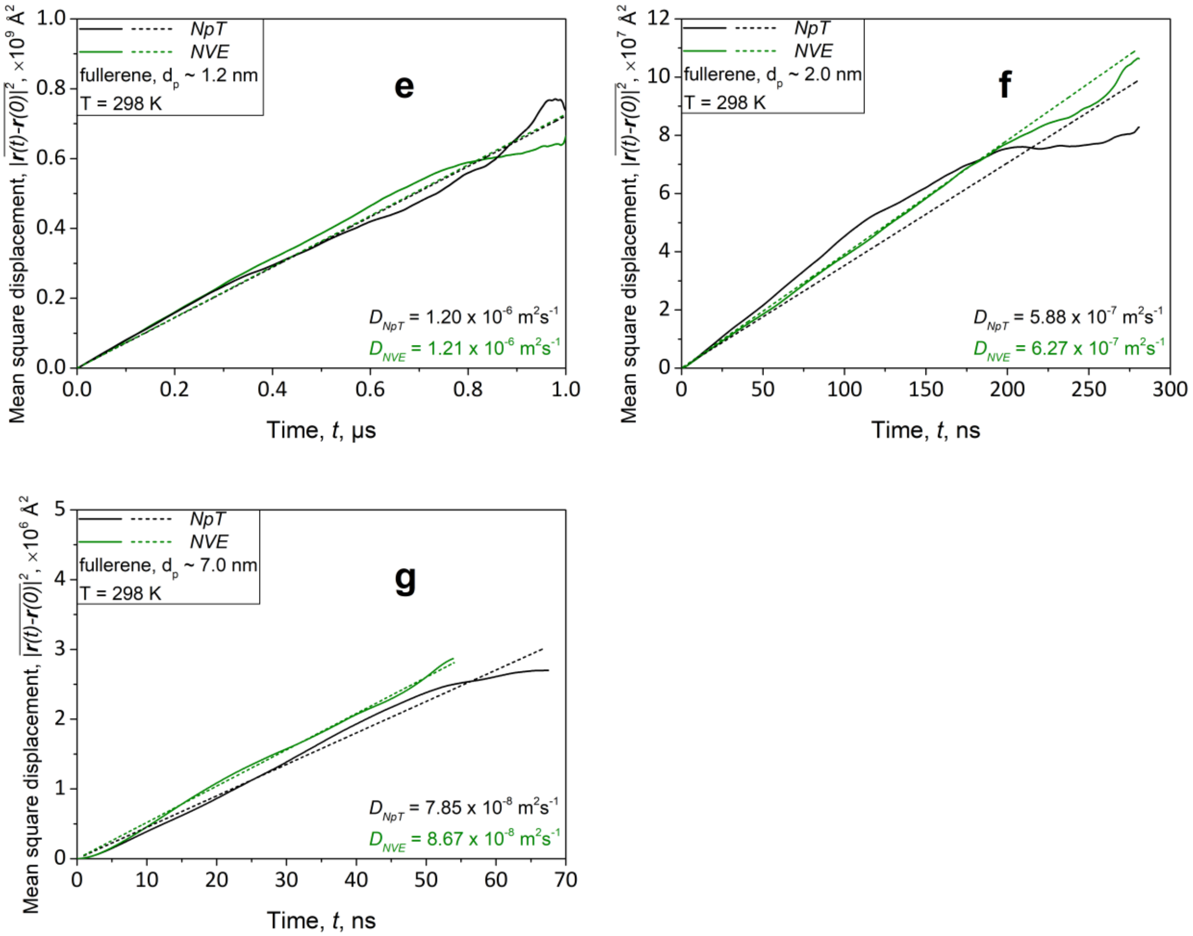

Figure S4. Mean square displacement (MSD) as a function of time for fullerene NPs with diameter a) 0.4, b) 0.5, c) 0.6, d) 0.7, e) 1.2, f) 2, and g) 7 nm from fully atomistic (FA) molecular dynamics (MD) simulations in *NpT* (black line), and *NVE* ensemble (green line). The broken lines correspond to fitting the above MD-obtained data with $MSD = 0 + 6 \cdot D \cdot t$. The diffusivities from both *NpT* and *NVE* ensembles are nearly identical (insets, Table S8 also).

Table S8. The diffusion coefficients for fullerene and silica NPs as calculated by *NpT* and *NVE* simulations are nearly identical.

d_p, nm	Type	D_{NpT}, m^{2}s^{-1}	D_{NVE}, m^{2}s^{-1}	Error, %
0.4	fullerene	5.07×10^{-6}	5.14×10^{-6}	1.5
0.5	fullerene	3.93×10^{-6}	3.38×10^{-6}	14
0.6	fullerene	3.04×10^{-6}	3.06×10^{-6}	0.7
0.7	fullerene	2.84×10^{-6}	3.09×10^{-6}	8.8
1.20	fullerene	1.20×10^{-6}	1.21×10^{-6}	0.7
2.0	fullerene	5.88×10^{-7}	6.27×10^{-7}	6.6
7.00	fullerene	7.85×10^{-8}	8.67×10^{-8}	10.4
1.5	silica	1.22×10^{-6}	1.27×10^{-6}	4.1
2.0	silica	6.24×10^{-7}	6.79×10^{-7}	8.8

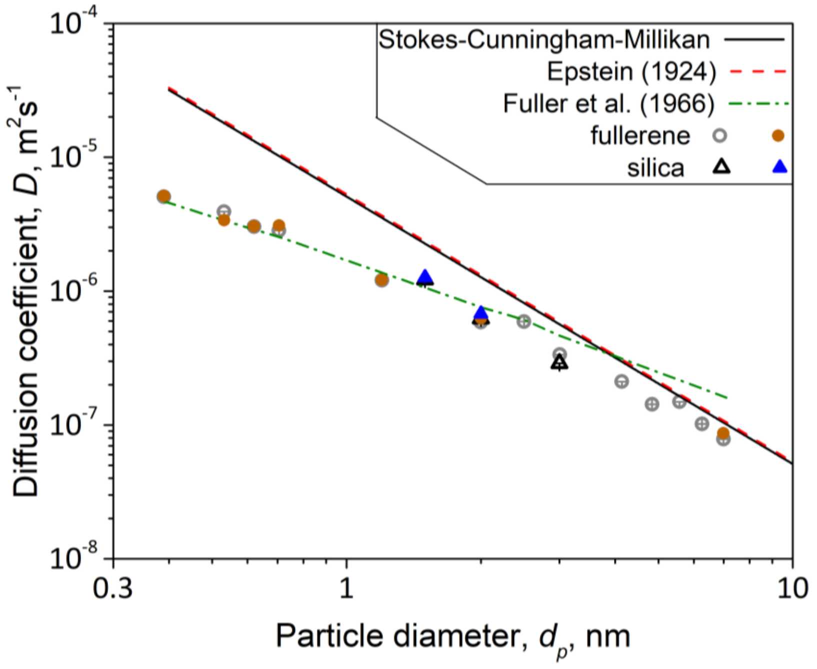

Figure S5. MD-obtained diffusion coefficients of fullerene (circles) and silica (triangles) NPs as a function of their d_p along with those from the Stokes-Cunningham-Millikan (solid black line) and Epstein (broken red line) equations (that totally overlap in this size regime) as well to that (dot-broken green line) by Fuller et al. and comparison between results obtained by employing the *NpT* ensemble (open symbols) and the *NVE* ensemble (filled symbols). The errors in the diffusion coefficients are smaller than the symbol size.

S3. DIFFUSIVITY CALCULATION AND COMPARISON WITH THEORETICAL MODELS

Table S9. Parameter values for calculation of diffusion coefficients for $C_{20}, D_{C_{20}}$, and $C_{60}, D_{C_{60}}$, fullerenes by Eq. 8 of Fuller et al.**[36]** at T = 298.15 K and p = 1 atm.

**Property**	**Value**	**Units**
$M_{\text{air,RH=0\%}}$	28.9647[1]	g mol^{-1}
M_{C}	12.0107	g mol^{-1}
$M_{\text{C}_{20}}$	$20 \times 12.0107 = 240.214$	g mol^{-1}
$M_{\text{C}_{60}}$	$60 \times 12.0107 = 720.642$	g mol^{-1}
$\sum_{i=1}^{3} V_{i,\text{air}}$	20.1[2]	Å^3
V_{C}	16.5[2]	Å^3
$V_{aromatic\ rings}$	-20.2[2]	Å^3
$\sum_{i=1}^{20} V_{i,\text{C}_{20}}$	$20 \times 16.5 - 20.2 = 309.8$	Å^3
$\sum_{i=1}^{60} V_{i,\text{C}_{60}}$	$60 \times 16.5 - 20.2 = 969.8$	Å^3
$D_{\text{C}_{20}}$	4.677×10^{-2}	cm^2 s^{-1}
$D_{\text{C}_{60}}$	2.547×10^{-2}	cm^2 s^{-1}

[1]https://www.engineeringtoolbox.com/air-composition-d_212.html

[2]Fuller et al. **[36]**

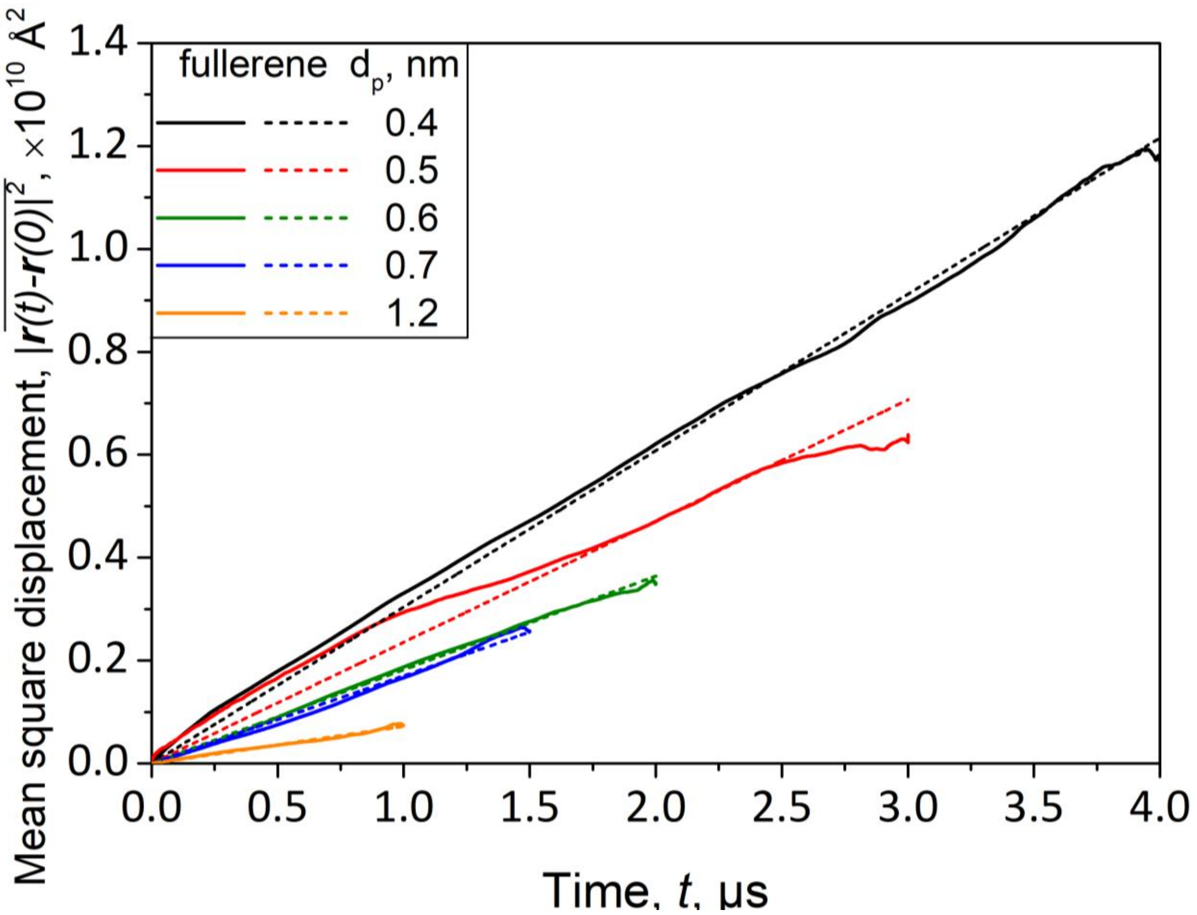

Figure S6. Mean square displacement (MSD, solid lines) as a function of time for fullerene NPs with diameter 0.4 (black), 0.5 (red), 0.6 (green), 0.7 (blue) and 1.2 nm (orange line) from fully atomistic (FA) molecular dynamics (MD) simulations. The broken lines correspond to fitting the above MD-obtained data with $MSD = 0 + 6 \cdot D \cdot t$, to extract the diffusion coefficients shown first in Fig. 3.

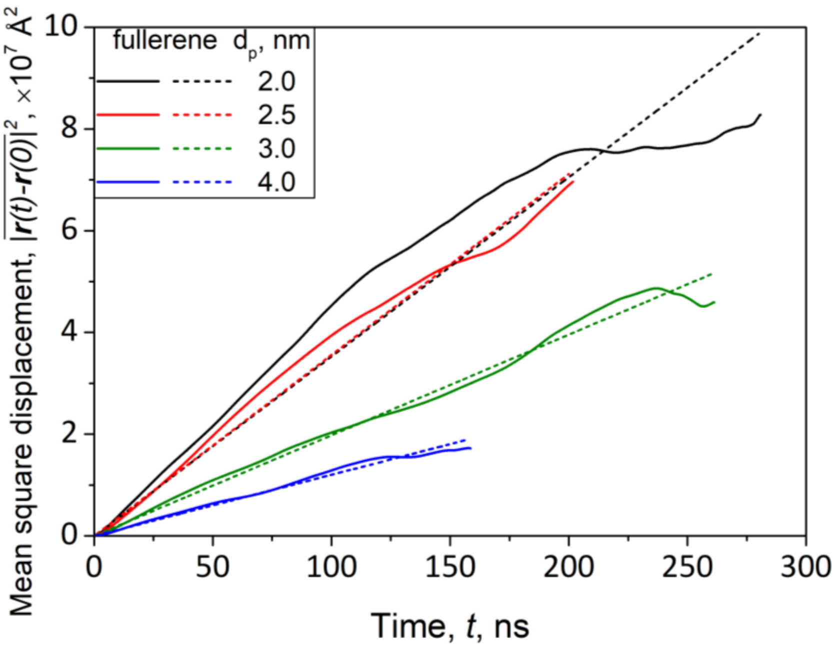

Figure S7. Mean square displacement (MSD, solid lines) as a function of time for fullerene NPs with diameter 2.0 (black), 2.5 (red), 3.0 (green), and 4.0 nm (blue line) from fully atomistic (FA) molecular dynamics (MD) simulations. The broken lines correspond to fitting the above MD-obtained data with $MSD = 0 + 6 \cdot D \cdot t$, to extract the diffusion coefficients shown first in Fig. 3.

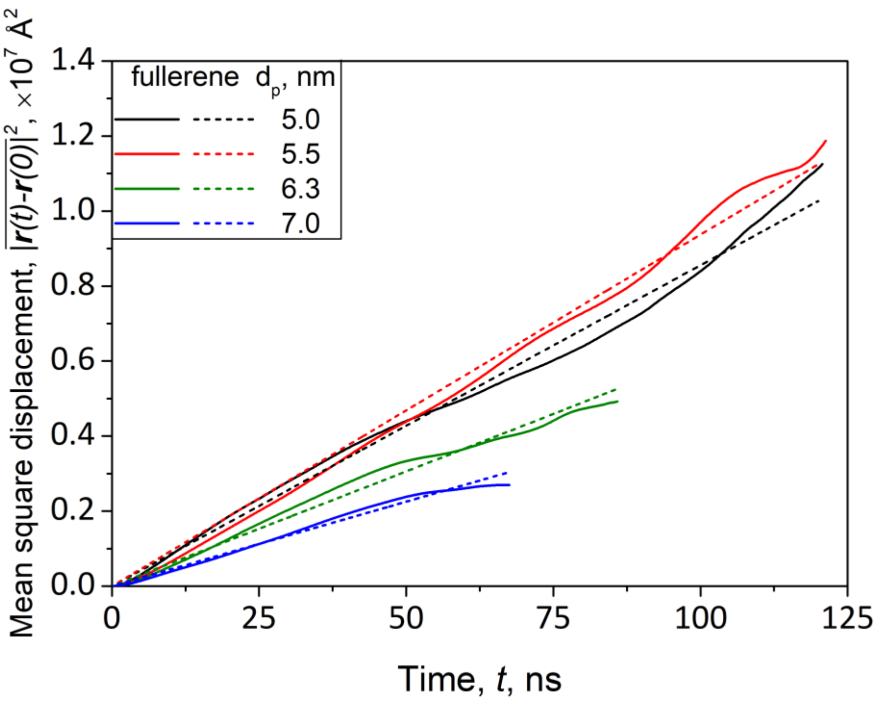

Figure S8. Mean square displacement (MSD, solid lines) as a function of time for fullerene NPs with diameter 4.8 (black), 5.5 (red), 6.3 (green), and 7.0 nm (blue line) from fully atomistic (FA) molecular dynamics (MD) simulations. The broken lines correspond to fitting the above MD-obtained data with $MSD = 0 + 6 \cdot D \cdot t$, to extract the diffusion coefficients shown first in Fig. 3.

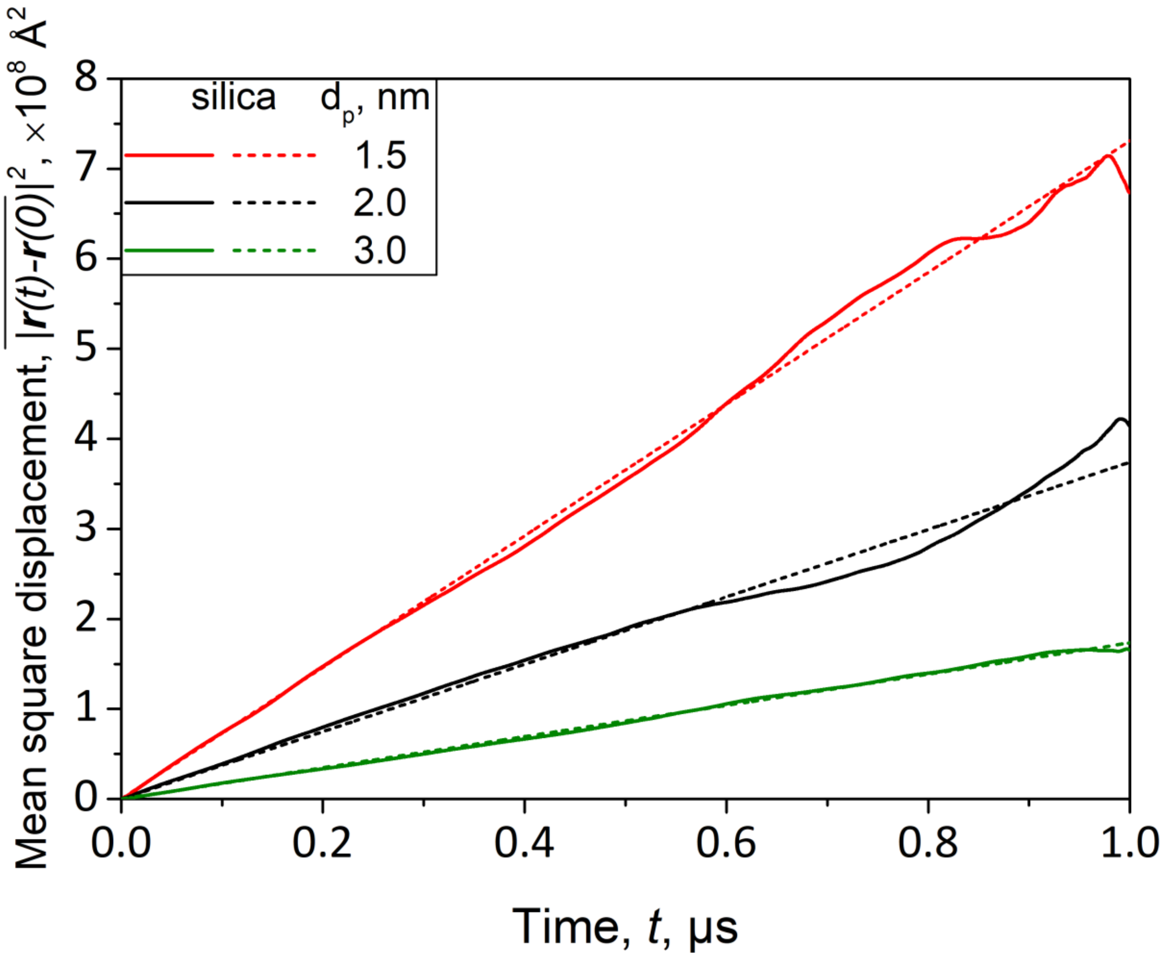

Figure S9. Mean square displacement (MSD, solid lines) as a function of time for silica NPs with diameter 1.5 (red), 2.0 (black), and 3.0 (green line) from fully atomistic (FA) molecular dynamics (MD) simulations. The broken lines correspond to fitting the above MD-obtained data with $MSD = 0 + 6 \cdot D \cdot t$, to extract the diffusion coefficients shown first in Fig. 3.

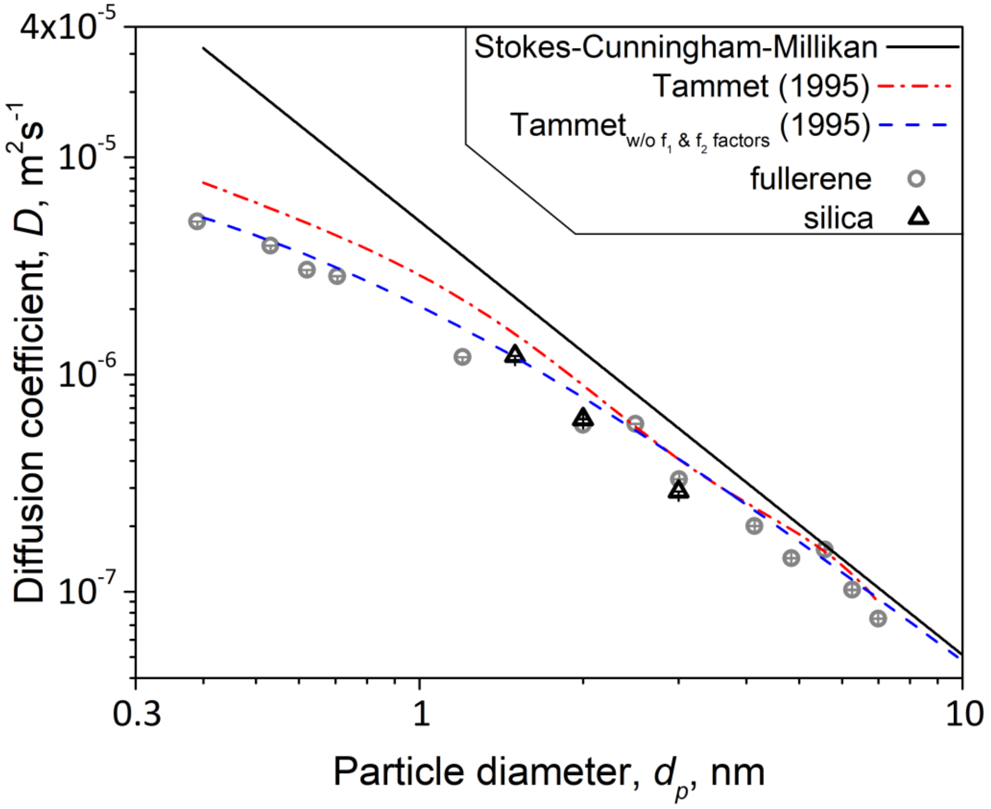

Figure S10. MD-obtained diffusivities of fullerene (circles) and silica (triangles) NPs in air at 298.15 K, as a function of their diameter along with predictions of the Stokes-Cunningham-Millikan Eq. 3 (solid line), Eq. 5 (dot-broken red line) by Tammet[7] and also by that equation but without (broken blue line) the terms proportional to the f_1 and f_2 factors.

S4. DESCRIPTION OF VIDEOS

Supplementary Video V1.mp4. Visualization of a grazing collision between a N_2 gas molecule (blue) and a C_{20} fullerene NP (cyan) in relative coordinates, with the reference (stationary) point being the center of mass of the fullerene.

Supplementary Video V2.mp4. An example of a nearly head-on collision between a N_2 gas molecule (blue) and a C_{20} fullerene NP (cyan) modeled in relative coordinates, where the NP's center of mass is considered as the reference (stationary) point.

Supplementary Video V3.mp4. Visualization of the trajectory of a 1.2 nm fullerene NP (orange) diffusing in air together with the paths of its center of mass (white line) and of an atom on its surface (grey line) depicting its translational and rotational motion, respectively. Also, selected air molecules are shown colliding with the NP, depicting grazing, multi-body and even orbitting[22] collisions of N_2 molecules with the NP.
